# Low urinary sodium-to-potassium ratio in the early phase following single-unit cord blood transplantation is a predictive factor for poor non-relapse mortality in adults

**DOI:** 10.1038/s41598-024-51748-7

**Published:** 2024-01-16

**Authors:** Kosuke Takano, Maki Monna-Oiwa, Masamichi Isobe, Seiko Kato, Satoshi Takahashi, Yasuhito Nannya, Takaaki Konuma

**Affiliations:** 1 Department of Hematology/Oncology, The Institute of Medical Science, The University of Tokyo, 4-6-1, Shirokanedai, Minato-ku, Tokyo, 108-8639 Japan; 2 Division of Clinical Precision Research Platform, The Institute of Medical Science, The University of Tokyo, Tokyo, Japan

**Keywords:** Haematological cancer, Prognostic markers

## Abstract

Although daily higher urinary sodium (Na) and potassium (K) excretion ratio is associated with the risk of cardiovascular disease in the general population, a low Na/K ratio is associated with renal dysfunction in critically ill patients. Thus, we retrospectively analyzed the impact of daily urinary Na and K excretion and their ratio on non-relapse mortality (NRM) and overall mortality in 172 adult single-unit cord blood transplantation (CBT) patients treated at our institution between 2007 and 2020. Multivariate analysis showed that a low urinary Na/K ratio at both 14 days (hazard ratio [HR], 4.82; 95% confidence interval [CI], 1.81–12.83; *P* = 0.001) and 28 days (HR, 4.47; 95% CI 1.32–15.12; *P* = 0.015) was significantly associated with higher NRM. Furthermore, a low urinary Na/K ratio at 28 days was significantly associated with higher overall mortality (HR, 2.38; 95% CI 1.15–4.91; *P* = 0.018). Patients with a low urinary Na/K ratio had decreased urine volume, more weight gain, experienced more grade III–IV acute graft-versus-host disease, and required corticosteroids by 28 days after CBT. These findings indicate that a low urinary Na/K ratio early after single-unit CBT is associated with poor NRM and survival in adults.

## Introduction

For adult patients without the appropriate matched related and unrelated donors, cord blood transplantation (CBT) serves as a practical alternative to unrelated allogeneic hematopoietic cell transplantation (HCT)^1–5^. However, persistent neutropenia and slow immunological recovery remain major drawbacks of single-unit CBT for adult patients, which may increase the risk of early infection problems and the need for potentially nephrotoxic medications after CBT. In addition, pre-engraftment syndrome (PES), a clinical manifestation characterized by high-grade fever, skin rash, and fluid retention, is another issue that arises after CBT^6–9^. These circumstances could contribute to renal hypoperfusion or dysfunction early after CBT.

Daily urinary sodium (Na) and potassium (K) excretion and their ratio are commonly used as surrogate markers for daily Na and K intake^10–15^. Among population-based epidemiological studies, a higher Na/K ratio in 24-h urine collection is associated with an increased risk of cardiovascular disease (CVD) via higher blood pressure, more so than daily urinary excretion of Na or K alone^14,15^. Thus, decreasing the daily urinary Na/K ratio is important for preventing CVD by reducing hypertension.

On the other hand, urinary Na or K excretion and Na/K ratio have been reported to be associated with renal dysfunction and mortality in critically ill patients or those with cirrhosis^16–23^. Among critically ill patients, lower urinary Na excretion^17,21^ and higher urinary K excretion^19,20^ were associated with renal dysfunction and predicted progress of acute kidney injury. Moreover, lower urinary Na/K ratio was associated with renal dysfunction and mortality among patients with cirrhosis^22,23^. However, there is a lack of research regarding the clinical significance of daily urinary Na and K excretion and their ratio in CBT recipients. As a result, we retrospectively analyzed whether daily urinary Na and K excretion and their ratio have an impact on post-transplant outcomes for adult patients who received single-unit CBT using cyclosporine (CSP)-based graft-versus-host disease (GVHD) prophylaxis in our institute.

## Results

### Patient characteristics

Table 1 presents the characteristics of patients, cord blood units, and transplantations. The median age of patients at CBT was 45.5 years, with a range of 16–69 years. Acute myeloid leukemia was the most common disease type, accounting for 49% of cases. The median cryopreserved cord blood total nucleated cell (TNC) dose was 2.58 × 10^7^/kg (range, 1.52–5.69 × 10^7^/kg). The median cryopreserved cord blood CD34 + cell dose was 1.03 × 10^5^/kg (range, 0.36–2.84 × 10^5^/kg). The number of HLA allele mismatches at HLA-A, -B, and -DRB1 between recipients and cord blood units was 0–2 in 81 (47%) patients and ≥ 3 in 91 (53%) patients. Among sex compatibility, 49 (28%) patients received a female donor to male recipient transplant. The majority of conditioning regimens were total body irradiation (TBI) 12 Gy, cyclophosphamide 120 mg/kg, and high-dose cytarabine 12 g/m^2^ (73%)^24,25^, followed by TBI 4 Gy, intravenous busulfan 9.6 mg/kg, fludarabine 180 mg/m^2^, and high-dose cytarabine 12 g/m^2^ (19%)^26^. The GVHD prophylaxis consisting of CSP and methotrexate (79%) was usually used for patients receiving TBI ≥ 10Gy-based conditioning regimens, whereas that consisting of CSP and mycophenolate mofetil (21%) was usually used for patients receiving the TBI 2-4Gy-based conditioning regimens. The median follow-up period for survivors was 74.4 months (range, 3.7–173.8 months).

**Table 1:** Patient characteristics.

Characteristic	Value
Number of patients	172
Median age at CBT, (range) years	45.5 (16–69)
Sex	
Male	108 (63%)
Female	64 (37%)
HCT-CI	
0–2	146 (85%)
≥ 3	26 (15%)
Diagnosis	
AML	84 (49%)
ALL	37 (22%)
MDS	26 (15%)
MPN	7 (4%)
NHL/ATL	7 (4%)
CML	6 (4%)
CAEBV/SAA	5 (3%)
Disease status*	
Standard-risk	84 (49%)
High-risk	83 (48%)
Not available	5 (3%)
Refined disease risk index	
Low/Intermediate	86 (52%)
High/Very high	80 (48%)
Not available	6
Cryopreserved cord blood TNC dose, (range) × 10^7^/kg	2.58 (1.52–5.69)
Cryopreserved cord blood CD34^+^ cell dose, (range) × 10^5^/kg	1.03 (0.36–2.84)
HLA disparities**	
0–2	81 (47%)
≥ 3	91 (53%)
Sex compatibility	
Female donor to male recipient	49 (28%)
Others	123 (72%)
Conditioning regimen	
TBI 12Gy + CY + HDCA ± G-CSF	126 (73%)
TBI12Gy + CY	6 (5%)
TBI10-12GY + CY + FLU	3 (2%)
TBI12Gy + FLU + MEL	1 (< 1%)
TBI4Gy + BU3 + FLU + HDCA ± G-CSF	32 (19%)
TBI4Gy + FLU + MEL	2 (1%)
TBI4Gy + FLU + HDCA + G-CSF	1 (< 1%)
GVHD prophylaxis	
CSP with MTX	135 (79%)
CSP with MMF	37 (21%)

*CBT* cord blood transplantation, *HCT-CI* hematopoietic cell transplantation specific comorbidity index, *AML* acute myeloid leukemia, *ALL* acute lymphoblastic leukemia, *MDS* myelodysplastic syndrome, *MPN* myeloproliferative neoplasm, *NHL* non-Hodgkin’s lymphoma, *ATL* adult T-cell leukemia, *CML* chronic myelogenous leukemia, *CAEBV* chronic active Epstein-Barr virus infection, *SAA* severe aplastic anemia, *TNC* total nucleated cell, *HLA* human leukocyte antigen, *TBI* total body irradiation, *CY* cyclophosphamide, *HDCA* high dose cytosine arabinoside, *G-CSF* granulocyte colony-stimulating factor, *FLU* fludarabine, *MEL* melphalan, *BU* busulfan, *GVHD* graft-versus-host disease, *CSP* cyclosporine, *MTX* methotrexate, *MMF* mycophenolate mofetil.

*For hematological malignancies, acute leukemia in first or second complete remission, CML in first chronic phase, NHL/ATL in complete or partial chemotherapy-sensitive remission, MDS, and MPN without excess blasts were considered standard-risk; all others were considered high-risk at the time of CBT.

**HLA disparities between cord blood graft and recipient were defined as a high-resolution for HLA-A, HLA-B, and HLA-DR.

Among the entire cohort, 161 patients achieved neutrophil recovery, with a median time to neutrophil engraftment (≥ 0.5 × 10^9^/L) of 21 days (range, 13–50 days). Among the 140, 165, and 163 patients assessable for PES, grade II-IV acute GVHD, and grade III–IV acute GVHD, the cumulative incidences of PES, grade II-IV acute GVHD, and grade III–IV acute GVHD at 100 days after CBT were 70.7% (95% confidence interval [CI], 62.3–77.6%), 78.2% (95% CI 71.0–83.8%), and 13.5% (95% CI 8.8–19.2%), respectively. The median times to the development of PES, grade II–IV acute GVHD, and grade III–IV acute GVHD were 14 days (range, 7–34 days), 19 days (range, 8–69 days), and 32 days (range, 9–49 days), respectively.

Among the entire cohort, 74 and 99 patients were treated with low-dose dopamine (2 μg/kg/min) with a median time of initiation of low-dose dopamine therapy at 9 days (range, 0–14 days) at 14 days, and 11 days (range, 0–28 days) days at 28 days after CBT, respectively. The median duration of use of low-dose dopamine was 5 days (interquartile range [IQR], 2–9 days), and 17 days (IQR, 13.5–21 days) at 14 days, and 28 days, respectively. Use of low-dose dopamine was associated with lower CrCl, lower serum Na and K levels, and higher serum creatinine levels at 14 days and 28 days (Supplementary Table 1).

### Characteristics of daily urinary Na and K excretion

The median urinary excretion of Na and K at day 14 was 114.40 mEq/day (IQR, 74.51–160.92 mEq/day) and 38.73 mEq/day (IQR, 28.11–49.99 mEq/day), respectively. The median urinary Na/K ratio at day 14 was 3.09 (IQR, 1.84–4.24).

**Figure 1:**
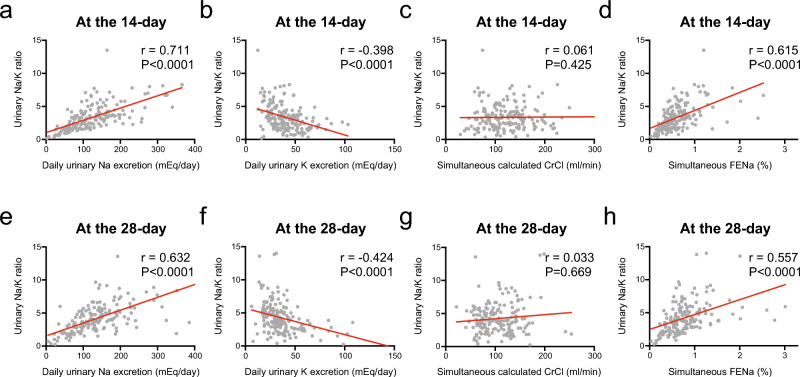
Correlation between daily urinary Na/K ratio and daily urinary Na excretion (**a**, **e**), daily urinary K excretion (**b**, **f**), simultaneous calculated creatinine clearance (CrCl) (**c**, **g**), and fractional excretion of sodium (FENa) (**d**, **h**) at 14 and 28 days post-CBT.

At day 14, there was a significant correlation between daily urinary Na/K ratio and daily urinary Na excretion (r = 0.711) (Fig. 1a), and fractional excretion of sodium (FENa) (r = 0.615) (Fig. 1d). Daily urinary Na/K ratio was weakly correlated with the urinary K excretion (r = − 0.398) (Fig. 1b). However, the daily urinary Na/K ratio was not correlated with creatine clearance (CrCl) (r = 0.061) (Fig. 1c). The correlation between daily urinary Na excretion and daily urinary K excretion (r = 0.299) and CrCl (r = 0.315) was weak. Daily urinary K excretion was weakly correlated with CrCl (r = 0.320).

Three patients died or developed anuria before the evaluation of daily urinary Na and K excretion at day 28. Among 169 evaluable patients, the median urinary Na and K excretion at day 28 was 128.90 mEq/day (IQR, 92.59–170.69 mEq/day) and 33.07 mEq/day (IQR, 25.63–40.91 mEq/day), respectively. The median urinary Na/K ratio at day 28 was 3.83 (IQR, 2.72–5.33).

At day 28, daily urinary Na/K ratio was weakly correlated with daily urinary Na excretion (r = 0.632) (Fig. 1e), daily urinary K excretion (r = − 0.424) (Fig. 1f), and FENa (r = 0.557) (Fig. 1h). However, the daily urinary Na/K ratio was not correlated with CrCl (r = 0.033) (Fig. 1g). Daily urinary Na excretion was weakly correlated with daily urinary K excretion (r = 0.348), and CrCl (r = 0.333). Daily urinary K excretion was also weakly correlated with CrCl (r = 0.307).

### Association between CSP trough levels and daily urinary Na and K excretion

**Figure 2:**
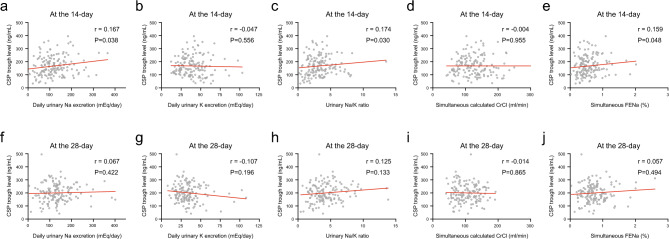
Correlation between CSP trough levels and daily urinary Na excretion (**a**, **f**), daily urinary K excretion (**b**, **g**), daily urinary Na/K ratio (**c**, **h**), simultaneous calculated creatinine clearance (CrCl) (**d**, **i**), and fractional excretion of sodium (FENa) (**e**, **j**) at 14 and 28 days post-CBT.

CSP trough levels were evaluated within 4 days at 14 days (n = 154) and 28 days (n = 145) after CBT. CSP trough levels were not correlated with daily urinary Na excretion (r = 0.167), daily urinary K excretion (r = − 0.047), daily urinary Na/K ratio (r = 0.174), CrCl (r = − 0.004), or FENa (r = 0.159) at day 14 (Fig. 2a–e). At day 28, CSP trough levels were not correlated with daily urinary Na excretion (r = 0.067), daily urinary K excretion (r = − 0.107), daily urinary Na/K ratio (r = 0.125), CrCl (r = − 0.014), or FENa (r = 0.057) at day 14 (Fig. 2f–j).

### Impact of daily urinary Na and K excretion on non-relapse mortality (NRM) and overall survival (OS)

The daily urinary excretion of Na at both 14 and 28 days did not display a correlation with NRM or OS in univariate (Supplementary Fig. 1) and multivariate analyses (Supplementary Table 2).

Regarding daily urinary K excretion at 14 and 28 days, univariate and multivariate analyses showed no association with NRM or OS, except for high urinary K excretion at 14 days (Supplementary Fig. 2, Supplementary Table 3), which was significantly associated with higher NRM in multivariate analysis (hazard ratio [HR] 2.99; 95% CI 1.02–8.72; *P* = 0.044) (Supplementary Table 3).

We also evaluated the impact of FENa on NRM and OS after CBT. The FENa at 14 and 28 days did not correlate with NRM or OS in univariate (Supplementary Fig. 3) and multivariate analyses (Supplementary Table 4).

### Impact of daily urinary Na/K ratio on NRM and OS

**Figure 3:**
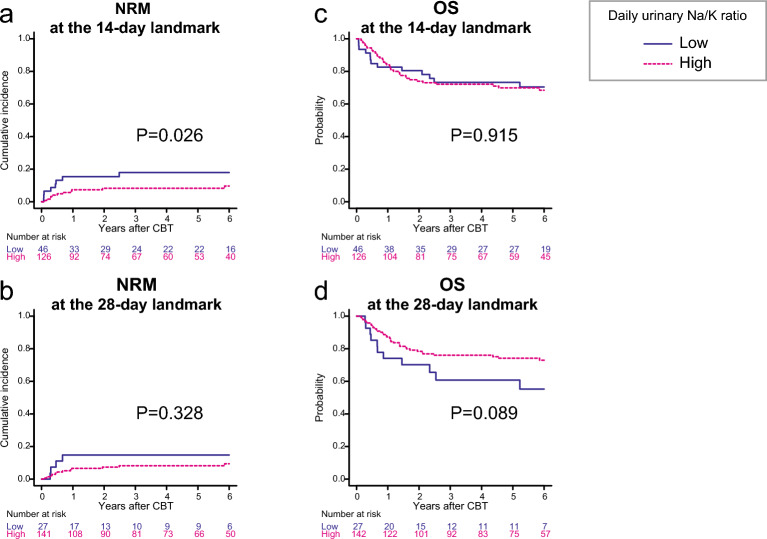
The effect of daily urinary Na/K ratio on non-relapse mortality (NRM) and overall survival (OS) following CBT. The cumulative incidence curves for NRM and Kaplan–Meier survival curves for OS based on daily urinary Na/K ratio were plotted, with a conditional landmark analysis conducted at both 14 (**a**, **c**) and 28 days (**b**, **d**) post-CBT. Three patients died or developed anuria before the evaluation of daily urinary Na and K excretion at day 28. In addition, one patient experienced relapse at 28 days.

In the univariate analysis, patients with a low urinary Na/K ratio at 14 days were significantly associated with higher NRM compared to those with a high urinary Na/K ratio at 14 days (*P* = 0.026) (Fig. 3a), but a low urinary Na/K ratio at 28 days was not associated with higher NRM (*P* = 0.328) (Fig. 3b). In multivariate analysis, both low urinary Na/K ratio at 14 days (HR, 4.82; 95% CI 1.81–12.83; *P* = 0.001) and at 28 days (HR, 4.47; 95% CI 1.32–15.12; *P* = 0.015) were significantly associated with higher NRM (Table 2).

**Table 2:** Multivariable analysis of non-relapse mortality and overall mortality for daily urinary Na/K ratio.

	Non-relapse mortality		Overall mortality	
	HR (95% CI)	*P-*value	HR (95% CI)	*P-*value
Landmark at 14 days				
Low daily urinary Na/K ratio at 14 days	4.82 (1.81–12.83)	**0.001**	1.59 (0.82–3.07)	0.163
Age ≥ 45 years	4.71 (1.22–18.16)	**0.024**	1.64 (0.85–3.17)	0.135
HCT-CI ≥ 3	1.10 (0.36–3.33)	0.859	0.84 (0.39–1.80)	0.666
High-risk disease status at CBT	1.51 (0.54–4.20)	0.427	2.33 (1.25–4.34)	**0.007**
Cord blood TNC ≥ 2.5 × 10^7^/kg	0.89 (0.35–2.23)	0.803	0.70 (0.39–1.24)	0.225
HLA disparities ≥ 3	1.49 (0.58–3.83)	0.399	1.06 (0.60–1.87)	0.826
Female donor to male recipient	4.12 (1.54–10.97)	**0.004**	2.20 (1.23–3.96)	**0.007**
TBI 2–4 Gy-based regimens	3.21 (1.05–9.80)	**0.004**	1.39 (0.68–2.83)	0.361
Landmark at 28 days				
Low daily urinary Na/K ratio at 14 days	4.47 (1.32–15.12)	**0.015**	2.38 (1.15–4.91)	**0.018**
Age ≥ 45 years	9.03 (1.84–44.38)	**0.006**	2.16 (1.07–4.36)	**0.030**
HCT-CI ≥ 3	1.57 (0.49–4.97)	0.441	0.91 (0.42–1.96)	0.819
High-risk disease status at CBT	1.00 (0.36–2.76)	0.986	1.91 (1.03–3.55)	**0.038**
Cord blood TNC ≥ 2.5 × 10^7^/kg	0.95 (0.35–2.54)	0.927	0.69 (0.39–1.24)	0.219
HLA disparities ≥ 3	1.88 (0.68–5.21)	0.220	1.09 (0.61–1.94)	0.767
Female donor to male recipient	2.47 (0.90–6.81)	0.078	1.94 (1.08–3.50)	**0.026**
TBI 2–4 Gy-based regimens	2.46 (0.77–7.80)	0.124	1.18 (0.56–2.48)	0.654

*Na/K* sodium-to-potassium, *HCT-CI* hematopoietic cell transplantation specific comorbidity index, *CBT* cord blood transplantation, *TNC* total nucleated cell, *HLA* human leukocyte antigen, *TBI* total body irradiation.

The *P*-values in bold are statistically significant (< 0.05).

In the univariate analysis, a low urinary Na/K ratio at 14 days (*P* = 0.915) (Fig. 3c) and 28 days (*P* = 0.089) (Fig. 3d) was not associated with lower OS. In multivariate analysis, a low urinary Na/K ratio at 28 days was significantly associated with higher overall mortality (HR, 2.38; 95% CI 1.15–4.91; *P* = 0.018), but a low urinary Na/K ratio at 14 days was not associated with overall mortality (HR, 1.59; 95% CI 0.82–3.07; *P* = 0.163) (Table 2).

### Clinical characteristics and parameters based on daily urinary Na/K ratio

Compared to patients with high urinary Na/K ratios at day 14, those with low urinary Na/K ratios at day 14 exhibited significantly reduced daily urine volume (*P* = 0.003), greater weight gain from baseline (*P* = 0.001), lower FENa (*P* < 0.001), and decreased serum Na levels (*P* = 0.039), and K levels (*P* < 0.001), as well as higher serum C-reactive protein (CRP) levels (*P* = 0.024) on the same day. (Table 3). The reduced urine volume, greater weight gain, and lower FENa could contribute to the significantly greater use of low-dose dopamine (*P* = 0.015), and higher serum CRP levels could be associated with a higher proportion of patients with high-grade fever (≥ 38.3 °C) (*P* = 0.015) (Table 3).

**Table 3:** Clinical parameters, complications, and administrated drugs based on daily urinary Na/K ratio.

	Urinary Na/K ratio at 14 days			Urinary Na/K ratio at 28 days *		
	Low	High	*P*-value	Low	High	*P*-value
Number of evaluable patients	46	126		27	142	
Parameters						
Urine volume, (IQR) ml/day	2898 (2611–3324)	3290 (2874–3845)	**0.003**	3010 (2315–3190)	3153 (2600–3639)	0.086
Rate of weight gain, (IQR) %	2.9 (0.6 to 4.8)	1.2 (− 1.1 to 2.8)	**0.001**	2.6 (− 1.7 to 5.8)	0.7 (− 1.5 to 3.0)	0.126
Creatinine clearance, (IQR) ml/min	105.7 (77.3–143.0)	122.1 (90.9–151.4)	0.128	115.4 (68.8–133.4)	102.0 (74.8–131.8)	0.792
FENa, (IQR) %	0.36 (0.21–0.49)	0.61 (0.45–0.82)	** < 0.001**	0.35 (0.24–0.58)	0.75 (0.58–1.04)	**< 0.001**
Serum albumin, (IQR) g/dL	2.9 (2.7–3.2)	3.1 (2.8–3.3)	0.102	3.3 (3.0–3.5)	3.2 (2.9–3.4)	0.424
Serum Na, (IQR) mEq/L	130.5 (126.0–134.0)	132.0 (129.0–135.0)	**0.039**	131.0 (127.0–133.5)	135.0 (132.0–137.0)	**0.001**
Serum K, (IQR) mEq/L	3.75 (3.50–4.10)	4.20 (3.80–4.50)	** < 0.001**	3.90 (3.65–4.25)	4.10 (3.80–4.50)	0.061
Serum CRP, (IQR) mg/dL	5.36 (2.36–9.35)	3.56 (1.53–6.85)	**0.024**	0.26 (0.14–1.27)	0.50 (0.22–1.43)	0.201
Complications						
Bacteremia	4 (8.7%)/46	18 (14.3%)/126	0.443	3 (11.1%)/27	19 (13.4%)/142	1.000
PES	20 (57.1%)/35	44 (41.9%)/105	0.123	19 (86.4%)/22	84 (73.0%)/125	0.281
High-grade fever (≥ 38.3 °C )	32 (69.6%)/46	60 (47.6%)/126	**0.015**	6 (22.2%)/27	24 (16.9%)/142	0.582
Grade II–IV aGVHD	14 (31.1%)/45	21 (17.5%)/120	0.085	14 (51.9%)/27	71 (52.2%)/136	1.000
Grade III–IV aGVHD	1 (2.3%)/44	2 (1.7%)/119	1.000	4 (14.8%)/27	4 (3.0%)/134	**0.028**
Administered drugs						
Diuretics	8 (17.4%)/46	22 (17.5%)/126	1.000	5 (18.5%)/27	42 (29.6%)/142	0.349
Low-dose dopamine	27 (58.7%)/46	47 (37.3%)/126	**0.015**	16 (59.3%)/27	80 (56.3%)/142	0.835
Corticosteroid 1–2 mg/kg/day	4 (8.7%)/46	5 (4.0%)/126	0.251	4 (14.8%)/27	9 (6.3%)/142	0.227
Corticosteroid 2 mg/kg/day	2 (4.3%)/46	2 (1.6%)/126	0.290	4 (14.8%)/27**	5 (3.5%)/142**	**0.037**

*IQR* interquartile range, *FENa* fractional excretion of sodium, *CRP* C-reactive protein, *PES* pre-engraftment syndrome, *aGVHD* acute graft-versus-host disease, *Na/K* sodium-to-potassium.

The *P* values in bold are statistically significant (< 0.05).

*Three patients died or developed anuria before the evaluation of daily urinary Na and K excretion at day 28.

**Among 9 evaluable patients who received a higher dosage of corticosteroids (2 mg/kg/day) by 28 days, 7 patients experienced grade III–IV acute GVHD by 28 days.

At day 28, patients with low urinary Na/K ratios had significantly lower serum Na levels on the same day (*P* = 0.001) and lower FENa (*P* < 0.001) compared to those with high urinary Na/K ratios (Table 3). Patients with a low urinary Na/K ratio at 28 days experienced more grade III–IV acute GVHD (*P* = 0.028), requiring a higher dosage of corticosteroids (2 mg/kg/day) by 28 days after CBT (*P* = 0.037) (Table 3).

Univariate analysis using a logistic regression model showed that high-grade fever (odd ratio [OD], 2.51; 95% CI 1.23–5.16; *P* = 0.011) and use of low-dose dopamine (OD, 2.39; 95% CI 1.20–4.76; *P* = 0.013) were significantly associated with low urinary Na/K ratios at 14 days. In multivariate analysis, use of low-dose dopamine maintained a significant association with low urinary Na/K ratios at 14 days (OD, 3.03; 95% CI 1.34–6.84; *P* = 0.007) (Table 4).

**Table 4:** Univariate and multivariate analyses of clinical factors associated with low urinary Na/K ratio at 14 days and 28 days.

	Univariate analysis		Multivariate analysis	
	Odd ratio (95%CI)	*P*	Odd ratio (95%CI)	*P*
At day 14				
Bacteremia	0.57 (0.18–1.79)	0.336		
PES	1.85 (0.85–4.01)	0.120		
High-grade fever (≥ 38.3 °C )	2.51 (1.23–5.16)	**0.011**		
Grade II–IV aGVHD	2.13 (0.96–4.68)	0.060		
Grade III–IV aGVHD	1.36 (0.12–15.40)	0.804		
Diuretics	0.99 (0.40–2.42)	0.992		
Low-dose dopamine	2.39 (1.20–4.76)	**0.013**	3.03 (1.34–6.84)	**0.007**
Corticosteroid 1–2 mg/kg/day	2.30 (0.59–8.99)	0.229		
Corticosteroid 2 mg/kg/day	2.82 (0.38–20.6)	0.307		
At day 28				
Bacteremia	0.80 (0.22–2.95)	0.748		
PES	2.34 (0.64–8.45)	0.195		
High-grade fever (≥ 38.3 °C )	1.40 (0.51–3.85)	0.509		
Grade II–IV aGVHD	0.98 (0.43–2.25)	0.973		
Grade III–IV aGVHD	5.65 (1.32–24.20)	**0.019**	5.47 (1.03–29.20)	**0.046**
Diuretics	0.54 (0.19–1.52)	0.245		
Low-dose dopamine	1.13 (0.48–2.60)	0.779		
Corticosteroid 1–2 mg/kg/day	2.57 (0.73–9.04)	0.141		
Corticosteroid 2 mg/kg/day	4.77 (1.19–19.10)	0.027		

*Na/K* sodium-to-potassium, *PES* pre-engraftment syndrome, *aGVHD* acute graft-versus-host disease, *CI* confidence interval.

The *P* values in bold are statistically significant (< 0.05).

At day 28, the development of grade III–IV acute GVHD was significantly associated with low urinary Na/K ratios at 28 days in univariate (OD, 5.65; 95% CI 1.32–24.20; *P* = 0.019) and multivariate (OD, 5.47; 95% CI 1.03–29.20; *P* = 0.046) analyses (Table 4).

### Cause of death

Among 172 patients, 53 patients died. The causes of death in patients based on daily urinary Na/K ratio at 14 days and at 28 days were shown in Table 5. Although the most common cause of death was relapse in both patient groups, the proportion of GVHD was a more common cause of death in patients with a low urinary Na/K ratio at 14 days and 28 days compared to those with a high urinary Na/K ratio at 14 days and 28 days, respectively (Table 5).

**Table 5:** Cause of death based on daily urinary Na/K ratio.

	Urinary Na/K ratio at 14 days		Urinary Na/K ratio at 28 days*	
	Low	High	Low	High
Number of deaths	15	38	11	39
Relapse	5 (33.3)	26 (68.4)	7 (63.6)	24 (61.5)
GVHD	6 (40.0)	6 (15.8)	4 (36.4)	7 (17.9)
Infection	2 (13.3)	1 (2.6)	0	1 (2.6)
Organ failure	1 (6.7)	4 (10.5)	0	5 (12.8)
Others	1 (6.7)	1 (2.6)	0	2 (5.1)

*GVHD* graft-versus-host disease, *Na/K* sodium-to-potassium.

*Three patients died or developed anuria before the evaluation of daily urinary Na and K excretion at day 28.

## Discussion

In the present study, we investigated the clinical significance of daily urinary excretion of Na and K and their ratio in the early phase after CBT in adults. Our findings demonstrate that a low daily urinary Na/K ratio at both 14 and 28 days after CBT is significantly associated with higher NRM, and a low urinary Na/K ratio at 28 days was significantly linked with higher overall mortality. Specifically, patients with a low urinary Na/K ratio at 14 days exhibit lower daily urine volume, more weight gain from baseline, lower FENa, lower serum Na and K levels, higher serum CRP levels, and a higher proportion of high-grade fever on the same day. Moreover, patients with a low urinary Na/K ratio at 28 days are more likely to develop grade III–IV acute GVHD. These findings suggest that the urinary Na/K ratio can predict NRM after CBT in adults, likely through the involvement of renal hypoperfusion.

In addition to being a surrogate marker for daily Na and K intake, the daily urinary Na/K ratio is also a marker of renal hypoperfusion and hyperaldosteronism^27,28^. Indeed, we found that patients with a low daily urinary Na/K ratio at 14 days exhibit a lower urine volume, higher rate of body weight gain, and lower FENa, compared to those with a high daily urinary Na/K ratio, indicating that a low daily urinary Na/K ratio seems to reflect renal hypoperfusion after CBT, which is consistent with previous studies showing that a low urinary Na/K ratio was associated with renal dysfunction and mortality in patients with cirrhosis^22,23^. Importantly, the daily urinary Na/K ratio was correlated with FENa, but not correlated with CrCl at both 14 days and 28 days, suggesting that the daily urinary Na/K ratio may serve as a surrogate marker for renal hypoperfusion. In addition, our previous study also demonstrated that the use of low-dose dopamine prevented body weight gain and the reduction of daily urine volume after CBT^29^, suggesting that low-dose dopamine could prevent the progression of renal hypoperfusion to renal dysfunction after CBT in the present study. Furthermore, we found that patients with a low daily urinary Na/K ratio at 28 days are more likely to develop grade III-IV acute GVHD related to the use of corticosteroid 2 mg/kg/day, compared to those with a high daily urinary Na/K ratio, indicating that a low daily urinary Na/K ratio seems to reflect hyperaldosteronism, in part due to the use of corticosteroids for the treatment of acute GVHD after CBT. These data suggest that the clinical findings in patients with a low daily urinary Na/K ratio could contribute to higher NRM after CBT.

Our data also showed that the daily urinary Na/K ratio at both 14 and 28 days is significantly associated with hyponatremia on the same day among CBT recipients. Hyponatremia has been reported to be the most common electrolyte imbalance in patients receiving allogeneic HCT^30^. The possible causes of hyponatremia after allogeneic HCT are likely multifactorial, including vomiting, diarrhea, intravenous fluids and medications, renal dysfunction, GVHD, sinusoidal obstruction syndrome, syndrome of inappropriate secretion of antidiuretic hormone, and human herpesvirus (HHV)-6 encephalitis^30–35^. Although CBT has been reported to be a risk factor for the development of HHV-6 encephalitis^36^, no patients in our cohort developed HHV-6 encephalitis. In contrast, our study clearly showed the association between the development of grade III–IV acute GVHD and the low daily Na/K ratio at 28 days after CBT. Therefore, future studies should clarify the association between posttransplant complications and urinary and serum electrolyte imbalances after allogeneic HCT, particularly CBT.

The present investigation is subject to certain limitations. Firstly, the retrospective single-center design of this study raises the possibility that local clinical practices may have influenced the study's outcomes. Secondly, the optimal timing and threshold for daily urinary Na and K excretion, as well as their ratio as a prognostic marker in patients receiving allogeneic HCT, remains a controversial topic. It is worth noting that Na and K intake targets may vary across different populations^37^. Furthermore, factors such as diet, intravenous fluids, blood transfusions, and medications could influence urinary Na and K excretion values among patients receiving CBT. Thirdly, our evaluation of daily urinary Na and K excretion values and their ratio relied on 24-h urine collection. However, several studies have shown that the mean Na/K ratio of repeated casual urine samples correlates similarly to that of 24-h urine collection among normotensive and hypertensive individuals^38,39^. Nonetheless, it remains unclear whether the daily urinary Na/K ratio using casual urine samples could predict NRM after CBT. Fourthly, we were unable to the association between the urinary Na/K ratio at 14 days and the development of PES, because patients with documented infection were excluded from the analysis evaluating their association due to the definition of PES^7^. Lastly, the exact mechanisms underlying the association between low daily urinary Na/K ratio and poor NRM have not been entirely elucidated. However, our data suggest that a low daily urinary Na/K ratio may serve as a surrogate marker for renal hypoperfusion and hyperaldosteronism, both of which may contribute to poor NRM in patients undergoing CBT.

In conclusion, our study demonstrates that a low urinary Na/K excretion ratio in the early stages following CBT is associated with inferior NRM and survival in adults, with a stronger correlation than with Na or K levels alone. While additional investigations are necessary to verify these findings, the urinary Na/K ratio in the early phases could potentially serve as a prognostic factor for the survival of adult patients undergoing single-unit CBT.

## Patients and methods

### Patient selection and transplant procedures

Between March 2007 and December 2020, this retrospective study included 175 successive adult patients who underwent single-unit CBT as their initial allogeneic HCT at our institute. Three patients who were not evaluated for 24-h urine volume and urine biochemistry at both 14 days and 28 days were excluded from this study. Finally, the remaining 172 patients were analyzed in this study. All cord blood units were obtained from cord blood banks in Japan, and the preferred unit was selected as reported previously^40^. Conditioning regimen, GVHD prophylaxis, and supportive care were decided by the treating physicians, as previously reported^24–26,41^. Low-dose dopamine (0.5–2 μg/kg/min) was used in our clinical practice to prevent and treat body weight gain, decreased urine volume, and/or decreased renal function^29^. Our study received approval from the Institutional Review Board of our institute (2021-110-0331). All methods were carried out in accordance with relevant guidelines and regulations.

### Urinary Na and K excretion and their ratio

We assessed 24-h urine volume and urine biochemistry, including measurements of electrolytes and creatinine, at least once a week during hospitalization for allogeneic HCT, following our standard practice. We calculated the total amount of urinary Na and K excreted in 24 h by multiplying the 24-h urine sample Na and K concentration (mEq/l) by the daily urine volume to give mEq/day of Na and K excreted in 24 h. CrCl was calculated using the standard formula: CrCl (mL/min) = urine volume (ml/min) × urinary creatinine concentration (mg/dl)/serum creatinine concentration (mg/dl), and was corrected for body surface area (BSA) using the formula: corrected CrCl = CrCl × (1.73 m^2^/BSA). BSA was calculated by the Du Bois formula. FENa was calculated by measuring creatinine and sodium levels in the blood and urine simultaneously: urinary sodium concentration (mEq/L) × serum creatinine concentration (mg/dl)/urinary creatinine concentration (mg/dl) × serum sodium concentration (mEq/L) × 100. Daily urinary excretion of Na and K, their ratio, CrCl, and FENa were calculated at 14 days and 28 days after CBT.

We determined the cutoff values for daily urinary Na and K excretion and their ratio from dietary Na and K intake based on the Dietary Reference Intakes for the Japanese, version 2020^42^. Considering that the daily Na intake was set to less than 2.95 g/day for males (128.4 mEq/day) and less than 2.56 g/day for females (111.3 mEq/day), daily urinary Na excretion of 128.4 mEq/day for males and 111.3 mEq/day was used as the cutoff value. The daily K intake was set to at least 3 g/day for males (76.7 mEq/day) and at least 2.6 g/day for females (66.5 mEq/day). Given that daily K intake was estimated from daily urinary K excretion by using a conversion factor of 1.3^43^, daily urinary K excretion of 59.0 mEq/day for males and 51.1 mEq/day was used as the cutoff value. The cutoff value for the daily urinary Na/K ratio was set to 2, which was previously indicated in previous reports^42,44^. The cutoff of 1% for FENa was used to define the sodium-avid state of prerenal azotemia (< 1%), or tubular damage and intrinsic kidney injury (≥ 1%)^45^.

### Measurement of CSP trough levels

The serum CSP trough levels were measured at our hospital using two techniques during this study period: the fluorescence polarization immunoassay (from March 2007 to February 2012) and the chemiluminescent immunoassay (from March 2012)^46^.

### Objective and definitions

This study aims to investigate the impact of daily urinary Na and K excretion and their ratio on NRM and OS after CBT for adults. NRM was defined as death during the remission of hematological disease as an indication for CBT. OS (inverse of overall mortality) was defined as the time between CBT and death or last contact. The impact of daily urinary Na and K excretion and their ratio on NRM and OS was evaluated using a landmark analysis at 14 and 28 days after CBT, as daily urinary Na and K excretion and their ratio were evaluated at 14 and 28 days after CBT. The disease status at CBT was classified as standard or high risk based on the risk scoring scheme of the American Society for Blood and Marrow Transplantation Request for Information 2006 risk scoring schema, as previously described^47^. The diagnosis of acute GVHD was based on previously established standard criteria^48^. PES was defined as an unexplained fever ≥ 38.3 °C not associated with documented infection and unresponsive to antimicrobial administrations and an unexplained erythematous skin rash resembling acute GVHD that occurred before neutrophil engraftment, as previously described^7^.

### Statistical analysis

The Spearman rank correlation coefficient was calculated to assess the correlation between daily urinary Na and K excretion, their ratio, simultaneous calculated CrCl, and simultaneous FENa.

The cumulative incidence method was employed to estimate the probability of NRM, while the Kaplan–Meier method was used to determine the probability of OS. Univariate analyses were performed using Gray’s test for NRM and a log-rank test for OS. For multivariate analysis, Cox proportional hazard models were used to investigate the main effects of daily urinary Na excretion (low vs. high), daily urinary K excretion (low vs. high), daily urinary Na/K ratio (low vs. high), or FENa (< 1% vs. ≥ 1%) on NRM and overall mortality (1-OS). The following possible confounding variables were also considered: recipient age (< 45 vs. ≥ 45 years), HCT-specific comorbidity index (0–2 vs. ≥ 3)^49^, disease status at CBT (high-risk vs. standard-risk)^47^, cryopreserved cord blood TNC count (< 2.5 vs. ≥ 2.5 × 10^7^/kg), HLA disparities defined as a high-resolution for HLA-A, -B, and -DRB1 (0–2 vs. ≥ 3), sex compatibility (female donor to male recipient vs. others) and conditioning regimen (TBI ≥ 10Gy-based regimens vs. TBI < 4Gy-based regimens). Recipient age and cord blood TNC were divided according to the approximate median value. The type of GVHD prophylaxis was not included in the variables of the multivariate analysis because selection for GVHD prophylaxis was associated with the type of conditioning regimen^24–26^. Group comparisons were conducted using the Mann–Whitney U test for continuous variables and Fisher's exact test for categorical variables.

Univariate and multivariate analyses were conducted to assess factors associated with low urinary Na/K ratios. Bacteremia, PES, high-grade fever, grade II–IV acute GVHD, grade III–IV acute GVHD, use of diuretics, use of low-dose dopamine, use of corticosteroid 1–2 mg/kg/day, and use of corticosteroid 2 mg/kg/day were included as factors and evaluated using a logistic regression model. The factors associated with low urinary Na/K ratios in the final model were confirmed with a backward selection procedure at a level of significance of *P* = 0.05.

The statistical analysis for the study was done using EZR (Saitama Medical Center, Jichi Medical University, Saitama, Japan)^50^, a graphical user interface for R 4.2.1 (The R Foundation for Statistical Computing, Vienna, Austria). Two-tailed *P*-values < 0.05 were considered to be significant.

### Ethical approval

Because this was a retrospective study, the opt-out method of obtaining informed consent was adopted. This retrospective study has been approved by the Institutional Review Board of the Institute of Medical Science, The University of Tokyo (2021-110-0331).

### Supplementary Information

Supplementary Information 1.

Supplementary Information 2.

Supplementary Information 3.

Supplementary Information 4.

Supplementary Information 5.

Supplementary Information 6.

Supplementary Information 7.

## Data Availability

The datasets used and/or analyzed during the current study are available from the corresponding author upon reasonable request.

## References

[1] Takahashi S (2004). Single-institute comparative analysis of unrelated bone marrow transplantation and cord blood transplantation for adult patients with hematologic malignancies. Blood.

[2] Takahashi S (2007). Comparative single-institute analysis of cord blood transplantation from unrelated donors with bone marrow or peripheral blood stem-cell transplants from related donors in adult patients with hematologic malignancies after myeloablative conditioning regimen. Blood.

[3] Shouval R (2019). Outcomes of allogeneic haematopoietic stem cell transplantation from HLA-matched and alternative donors: A European Society for Blood and Marrow Transplantation registry retrospective analysis. Lancet Haematol..

[4] Miyao K (2020). Updated comparison of 7/8 HLA allele-matched unrelated bone marrow transplantation and single-unit umbilical cord blood transplantation as alternative donors in adults with acute leukemia. Biol. Blood Marrow Transplant..

[5] Konuma T (2021). Single cord blood transplantation versus unmanipulated haploidentical transplantation for adults with acute myeloid leukemia in complete remission. Transplant. Cell. Ther..

[6] Kishi Y (2005). Early immune reaction after reduced-intensity cord-blood transplantation for adult patients. Transplantation.

[7] Patel KJ (2010). Pre-engraftment syndrome after double-unit cord blood transplantation: A distinct syndrome not associated with acute graft-versus-host disease. Biol. Blood Marrow Transplant..

[8] Park M (2013). Pre-engraftment syndrome after unrelated cord blood transplantation: A predictor of engraftment and acute graft-versus-host disease. Biol Blood Marrow Transplant..

[9] Konuma T (2017). Cytokine profiles of pre-engraftment syndrome after single-unit cord blood transplantation for adult patients. Biol. Blood Marrow Transplant..

[10] O'Donnell MJ (2011). Urinary sodium and potassium excretion and risk of cardiovascular events. JAMA.

[11] O'Donnell M (2014). Urinary sodium and potassium excretion, mortality, and cardiovascular events. N. Engl. J. Med..

[12] Mente A (2018). Urinary sodium excretion, blood pressure, cardiovascular disease, and mortality: A community-level prospective epidemiological cohort study. Lancet.

[13] Ma Y (2022). 24-Hour urinary sodium and potassium excretion and cardiovascular risk. N. Engl. J. Med..

[14] Intersalt: An international study of electrolyte excretion and blood pressure. Results for 24 hour urinary sodium and potassium excretion. Intersalt Cooperative Research Group. *BMJ*. **297**, 319–328 (1988). 10.1136/bmj.297.6644.319 PMC1834069 3416162

[15] Cook NR (2009). Joint effects of sodium and potassium intake on subsequent cardiovascular disease: The Trials of Hypertension Prevention follow-up study. Arch. Intern. Med..

[16] Pépin MN, Bouchard J, Legault L, Ethier J (2007). Diagnostic performance of fractional excretion of urea and fractional excretion of sodium in the evaluations of patients with acute kidney injury with or without diuretic treatment. Am. J. Kidney Dis..

[17] Maciel AT, Park M, Macedo E (2013). Physicochemical analysis of blood and urine in the course of acute kidney injury in critically ill patients: A prospective, observational study. BMC Anesthesiol..

[18] Maciel AT, Vitorio D, Salles LD, Park M (2014). Sodium concentration in urine greater than in the plasma: Possible biomarker of normal renal function and better outcome in critically ill patients. Anaesth. Intensive Care.

[19] Burns AR, Ho KM (2018). Urinary potassium excretion and its association with acute kidney injury in the intensive care unit. J. Crit. Care.

[20] Kumar NS (2021). Association between urinary potassium excretion and acute kidney injury in critically ill patients. Indian J. Crit. Care Med..

[21] de Morais DG (2022). Urinary sodium excretion is low prior to acute kidney injury in patients in the intensive care unit. Front. Nephrol..

[22] Cholongitas E (2013). Association between ratio of sodium to potassium in random urine samples and renal dysfunction and mortality in patients with decompensated cirrhosis. Clin. Gastroenterol. Hepatol..

[23] Morais Rateke EC (2021). Low sodium to potassium ratio in spot urine sample is associated with progression to acute kidney injury and mortality in hospitalized patients with cirrhosis. Dig. Liver Dis..

[24] Konuma T (2014). Single-unit cord blood transplantation after granulocyte colony-stimulating factor-combined myeloablative conditioning for myeloid malignancies not in remission. Biol. Blood Marrow Transplant..

[25] Konuma T, Kato S, Oiwa-Monna M, Tojo A, Takahashi S (2015). Single-unit cord blood transplant for acute lymphoblastic leukemia and lymphoma using an intensified conditioning regimen of total body irradiation, high-dose cytarabine and cyclophosphamide. Leuk. Lymphoma.

[26] Konuma T (2019). Reduced-Toxicity Myeloablative Conditioning Consisting of Fludarabine/Busulfan/Low-Dose Total Body Irradiation/Granulocyte Colony-Stimulating Factor-Combined Cytarabine in Single Cord Blood Transplantation for Elderly Patients with Nonremission Myeloid Malignancies. Biol. Blood Marrow Transplant..

[27] Wołyniec W, Kasprowicz K, Rita-Tkachenko P, Renke M, Ratkowski W (2019). Biochemical markers of renal hypoperfusion, hemoconcentration, and proteinuria after extreme physical exercise. Medicina (Kaunas).

[28] Segawa H (2021). Urinary sodium/potassium ratio as a screening tool for hyperaldosteronism in men with hypertension. Hypertens. Res..

[29] Konuma T (2019). Early fluid overload predicts higher non-relapse and overall mortality in adults after single-unit cord blood transplantation. Bone Marrow Transplant..

[30] Lee JH (2005). Severe metabolic abnormalities after allogeneic hematopoietic cell transplantation. Bone Marrow Transplant..

[31] Koumpis E, Florentin M, Hatzimichael E, Liamis G (2020). Hyponatremia in patients with hematologic diseases. J. Clin. Med..

[32] Kobayashi R (2004). Hyponatremia and syndrome of inappropriate antidiuretic hormone secretion complicating stem cell transplantation. Bone Marrow Transplant..

[33] Suzuki Y (2008). The syndrome of inappropriate secretion of antidiuretic hormone associated with SCT: Clinical differences following SCT using cord blood and BM/peripheral blood. Bone Marrow Transplant..

[34] Murakami K (2017). Hyponatremia associated with human herpesvirus-6 (HHV-6) encephalitis after allogeneic hematopoietic stem cell transplantation: A presentation different from HHV-6 myelitis. Int. J. Hematol..

[35] Yoshida S (2023). The preceding hyponatremia is a useful hallmark for the diagnosis of HHV-6 encephalitis after allogeneic hematopoietic stem cell transplantation. Bone Marrow Transplant..

[36] Ogata M (2017). Clinical characteristics and outcome of human herpesvirus-6 encephalitis after allogeneic hematopoietic stem cell transplantation. Bone Marrow Transplant..

[37] Drewnowski A, Rehm CD, Maillot M, Mendoza A, Monsivais P (2015). The feasibility of meeting the WHO guidelines for sodium and potassium: A cross-national comparison study. BMJ Open.

[38] Iwahori T (2014). Six random specimens of daytime casual urine on different days are sufficient to estimate daily sodium/potassium ratio in comparison to 7-day 24-h urine collections. Hypertens. Res..

[39] Iwahori T (2016). Four to seven random casual urine specimens are sufficient to estimate 24-h urinary sodium/potassium ratio in individuals with high blood pressure. J. Hum. Hypertens..

[40] Konuma T (2017). Cryopreserved CD34^+^ cell dose, but not total nucleated cell dose, influences hematopoietic recovery and extensive chronic graft-versus-host disease after single-unit cord blood transplantation in adult patients. Biol. Blood Marrow Transplant..

[41] Mizusawa M (2020). Clinical outcomes of persistent colonization with multidrug-resistant Gram-negative rods in adult patients undergoing single cord blood transplantation. Int. J. Hematol..

[42] Salman E (2022). Investigation of the urinary sodium-to-potassium ratio target level based on the recommended dietary intake goals for the Japanese population: The INTERMAP Japan. Hypertens. Res..

[43] Aburto NJ (2013). Effect of increased potassium intake on cardiovascular risk factors and disease: Systematic review and meta-analyses. BMJ.

[44] Kogure M (2022). Consideration of the reference value and number of measurements of the urinary sodium-to-potassium ratio based on the prevalence of untreated home hypertension: TMM Cohort Study. Hypertens. Res..

[45] Diamond JR, Yoburn DC (1982). Nonoliguric acute renal failure associated with a low fractional excretion of sodium. Ann. Intern. Med..

[46] Konuma T (2023). Clinical implications of augmented renal clearance after unrelated single cord blood transplantation in adults. Int. J. Hematol..

[47] Ross JA (2015). An exploratory analysis of mitochondrial haplotypes and allogeneic hematopoietic cell transplantation outcomes. Biol. Blood Marrow Transplant..

[48] Przepiorka, D. *et al*. 1994 Consensus conference on acute GVHD grading. *Bone Marrow Transplant*. **15**, 825–828 (1995). 7581076

[49] Sorror ML (2005). Hematopoietic cell transplantation (HCT)-specific comorbidity index: A new tool for risk assessment before allogeneic HCT. Blood.

[50] Kanda Y (2013). Investigation of the freely available easy-to-use software 'EZR' for medical statistics. Bone Marrow Transplant..

