# Supplementary material for: Low urinary sodium-to-potassium ratio in the early phase following single-unit cord blood transplantation is a predictive factor for poor non-relapse mortality in adults
Source: Sci Rep. 2024 Jan 16;14:1413. doi: 10.1038/s41598-024-51748-7 (PMC10791692; doi:10.1038/s41598-024-51748-7)
Supplement: Supplementary file 4 — Supplementary Information 4. [file 41598_2024_51748_MOESM4_ESM.docx]

**Supplementary Table 1.** Clinical data based on the use of low-dose dopamine.

|  | Use of low-dose dopamine at 14 days |  |  | Use of low-dose dopamine at 28 days * |  |  |
| --- | --- | --- | --- | --- | --- | --- |
|  | No use | Use | *P*-value | No use | Use | *P*-value |
| Number of evaluable patients | 98 | 74 |  | 73 | 96 |  |
| Parameters |  |  |  |  |  |  |
| Urine volume, (IQR) ml/day | 3105 (2697-3729) | 3302 (2854-3724) | 0.187 | 3084 (2557-3550) | 3159 (2600-3731) | 0.138 |
| Urine Na concentration, (IQR) mEq/L | 37 (31-51.5) | 32.5 (22-44) | **0.022** | 47 (32-58) | 39 (26-53) | 0.104 |
| Urine K concentration, (IQR) mEq/L | 13 (9-15) | 12 (10-17) | 0.518 | 11 (8-15) | 11 (9-13) | 0.433 |
| Urine creatinine concentration (IQR) mg/dL | 32 (24-43) | 30 (23-40.5) | 0.239 | 32 (25.8-42.5) | 28 (23-35) | **0.025** |
| Daily urinary Na excretion, (IQR) mEq/day | 119.52 (85.37-161.97) | 100.50 (63.17-156.93) | 0.126 | 133.40 (96.91-182.40) | 124.88 (85.92-156.70) | 0.306 |
| Daily urinary K excretion, (IQR) mEq/day | 38.18 (27.49-47.18) | 39.23 (30.45-53.16) | 0.074 | 32.40 (24.85-41.65) | 33.12 (26.44-39.95) | 0.967 |
| Daily urinary Na/K ratio, (IQR) | 3.27 (2.26-4.39) | 2.46 (1.63-3.88) | **0.027** | 4.00 (2.73-5.43) | 3.75 (2.70-5.20) | 0.510 |
| Creatinine clearance, (IQR) ml/min | 129.5 (102.2-157.2) | 102.0 (79.9-139.0) | **0.002** | 113.2 (90.4-142.0) | 87.9 (68.3-124.0) | **0.001** |
| FENa, (IQR) % | 0.53 (0.03-1.30) | 0.52 (0.11-2.53) | 0.779 | 0.66 (0.51-0.83) | 0.76 (0.55-1.04) | **0.085** |
| Serum Na, (IQR) mEq/L | 133(130-136) | 130 (126-133) | **<0.001** | 135.0 (132.0-138.0) | 133.0 (130.0-136.0) | **0.032** |
| Serum K, (IQR) mEq/L | 4.20 (3.90-4.57) | 3.70 (3.50-4.20) | **<0.001** | 4.20 (3.90-4.50) | 4.00 (3.70-4.40) | 0.053 |
| Serum creatine, (IQR) mg/dL | 0.60 (0.47-0.73) | 0.70 (0.54-0.82) | **0.003** | 0.60 (0.50-0.80) | 0.70 (0.58-0.89) | **0.026** |

IQR, interquartile range; FENa, fractional excretion of sodium; Na/K, sodium-to-potassium.

The *P* values in bold are statistically significant (<0.05).

* Three patients died or developed anuria before the evaluation of daily urinary Na and K excretion at day 28.
