# Supplementary material for: Low urinary sodium-to-potassium ratio in the early phase following single-unit cord blood transplantation is a predictive factor for poor non-relapse mortality in adults
Source: Sci Rep. 2024 Jan 16;14:1413. doi: 10.1038/s41598-024-51748-7 (PMC10791692; doi:10.1038/s41598-024-51748-7)
Supplement: Supplementary file 5 — Supplementary Information 5. [file 41598_2024_51748_MOESM5_ESM.docx]

**Supplementary Table 2**. Multivariable analysis of non-relapse mortality and overall mortality for daily urinary Na excretion.

|  | Non-relapse mortality |  | Overall mortality |  |
| --- | --- | --- | --- | --- |
|  | HR (95% CI) | *P-*value | HR (95% CI) | *P-*value |
| Landmark at 14 days |  |  |  |  |
| Low daily urinary Na excretion at 14 days | 1.32 (0.53-3.29) | 0.545 | 1.08 (0.61-1.89) | 0.781 |
| Age ≥ 45 years | 4.05 (1.07-15.27) | **0.038** | 1.62 (0.84-3.12) | 0.144 |
| HCT-CI ≥ 3 | 1.12 (0.36-3.43) | 0.834 | 0.85 (0.39-1.82) | 0.678 |
| High-risk disease status at CBT | 1.36 (0.52-3.58) | 0.522 | 2.22 (1.20-4.08) | **0.010** |
| Cord blood TNC ≥ 2.5 × 10^7^ /kg | 1.13 (0.46-2.74) | 0.784 | 0.75 (0.43-1.31) | 0.314 |
| HLA disparities ≥ 3 | 1.61 (0.64-4.01) | 0.305 | 1.13 (0.65-1.97) | 0.651 |
| Female donor to male recipient | 2.65 (1.07-6.53) | **0.033** | 2.03 (1.15-3.58) | **0.014** |
| TBI 2-4 Gy-based regimens | 2.71 (0.95-7.73) | 0.061 | 1.37 (0.68-2.79) | 0.372 |
| Landmark at 28 days |  |  |  |  |
| Low daily urinary Na excretion at 28 days | 0.91 (0.33-2.47) | 0.854 | 1.17 (0.65-2.11) | 0.586 |
| Age ≥ 45 years | 6.73 (1.42-31.94) | **0.016** | 1.74 (0.89-3.38) | 0.100 |
| HCT-CI ≥ 3 | 1.35 (0.43-4.22) | 0.604 | 0.90 (0.42-1.94) | 0.803 |
| High-risk disease status at CBT | 1.00 (0.37-2.74) | 0.987 | 2.00 (1.08-3.71) | **0.027** |
| Cord blood TNC ≥ 2.5 × 10^7^ /kg | 0.99 (0.38-2.59) | 0.998 | 0.69 (0.39-1.23) | 0.218 |
| HLA disparities ≥ 3 | 2.00 (0.71-5.65) | 0.188 | 1.14 (0.63-2.05) | 0.660 |
| Female donor to male recipient | 2.09 (0.78-5.62) | 0.141 | 1.85 (1.03-3.32) | **0.038** |
| TBI 2-4 Gy-based regimens | 2.21 (0.71-6.87) | 0.171 | 1.22 (0.58-2.57) | 0.585 |

Na, sodium; HCT-CI, hematopoietic cell transplantation comorbidity index; CBT, cord blood transplantation; TNC, total nucleated cell; HLA, human leukocyte antigen; TBI, total body irradiation.

The *P*-values in bold are statistically significant (<0.05).
