# Supplementary material for: Low urinary sodium-to-potassium ratio in the early phase following single-unit cord blood transplantation is a predictive factor for poor non-relapse mortality in adults
Source: Sci Rep. 2024 Jan 16;14:1413. doi: 10.1038/s41598-024-51748-7 (PMC10791692; doi:10.1038/s41598-024-51748-7)
Supplement: Supplementary file 6 — Supplementary Information 6. [file 41598_2024_51748_MOESM6_ESM.docx]

**Supplementary Table 3**. Multivariable analysis of non-relapse mortality and overall mortality for daily urinary K excretion.

|  | Non-relapse mortality |  | Overall mortality |  |
| --- | --- | --- | --- | --- |
|  | HR (95% CI) | *P-*value | HR (95% CI) | *P-*value |
| Landmark at 14 days |  |  |  |  |
| High daily urinary K excretion at 14 days | 2.99 (1.02-8.72) | **0.044** | 1.23 (0.56-2.66) | 0.597 |
| Age ≥ 45 years | 4.27 (1.12-16.16) | **0.032** | 1.61 (0.84-3.10) | 0.148 |
| HCT-CI ≥ 3 | 1.20 (0.40-3.64) | 0.738 | 0.87 (0.40-1.86) | 0.721 |
| High-risk disease status at CBT | 1.29 (0.48-3.49) | 0.605 | 2.19 (1.19-4.04) | **0.011** |
| Cord blood TNC ≥ 2.5 × 10^7^ /kg | 1.00 (0.40-2.49) | 0.985 | 0.73 (0.41-1.29) | 0.281 |
| HLA disparities ≥ 3 | 1.45 (0.58-3.61) | 0.424 | 1.11 (0.63-1.95) | 0.706 |
| Female donor to male recipient | 3.43 (1.30-9.07) | **0.012** | 2.08 (1.16-3.73) | **0.013** |
| TBI 2-4 Gy-based regimens | 2.74 (0.95-7.85) | 0.059 | 1.37 (0.67-2.77) | 0.376 |
| Landmark at 28 days |  |  |  |  |
| High daily urinary K excretion at 28 days | 1.63 (0.33-7.90) | 0.540 | 1.11 (0.38-3.18) | 0.843 |
| Age ≥ 45 years | 6.87 (1.45-32.42) | **0.014** | 1.78 (0.91-3.47) | 0.089 |
| HCT-CI ≥ 3 | 1.35 (0.43-4.23) | 0.596 | 0.91 (0.42-1.95) | 0.813 |
| High-risk disease status at CBT | 1.02 (0.37-2.78) | 0.968 | 2.03 (1.10-3.75) | **0.023** |
| Cord blood TNC ≥ 2.5 × 10^7^ /kg | 0.96 (0.36-2.51) | 0.938 | 0.68 (0.38-1.21) | 0.200 |
| HLA disparities ≥ 3 | 1.91 (0.69-5.27) | 0.210 | 1.18 (0.66-2.09) | 0.571 |
| Female donor to male recipient | 2.20 (0.80-6.01) | 0.124 | 1.85 (1.03-3.31) | **0.039** |
| TBI 2-4 Gy-based regimens | 2.12 (0.67-6.67) | 0.196 | 1.18 (0.56-2.48) | 0.659 |

K, potassium; HCT-CI, hematopoietic cell transplantation comorbidity index; CBT, cord blood transplantation; TNC, total nucleated cell; HLA, human leukocyte antigen; TBI, total body irradiation.

The *P*-values in bold are statistically significant (<0.05).
