# Supplementary material for: Low urinary sodium-to-potassium ratio in the early phase following single-unit cord blood transplantation is a predictive factor for poor non-relapse mortality in adults
Source: Sci Rep. 2024 Jan 16;14:1413. doi: 10.1038/s41598-024-51748-7 (PMC10791692; doi:10.1038/s41598-024-51748-7)
Supplement: Supplementary file 7 — Supplementary Information 7. [file 41598_2024_51748_MOESM7_ESM.docx]

**Supplementary Table 4**. Multivariable analysis of non-relapse mortality and overall mortality for FENa.

|  | Non-relapse mortality |  | Overall mortality |  |
| --- | --- | --- | --- | --- |
|  | HR (95% CI) | *P-*value | HR (95% CI) | *P-*value |
| Landmark at 14 days |  |  |  |  |
| High FENa (≥ 1%) at 14 days | 0.87 (0.24-3.06) | 0.830 | 0.75 (0.29-1.94) | 0.564 |
| Age ≥ 45 years | 4.07 (1.07-15.43) | **0.038** | 1.69 (0.87-3.29) | 0.118 |
| HCT-CI ≥ 3 | 1.14 (0.37-3.46) | 0.816 | 0.86 (0.40-1.85) | 0.714 |
| High-risk disease status at CBT | 1.29 (0.49-3.41) | 0.600 | 2.23 (1.19-4.15) | **0.011** |
| Cord blood TNC ≥ 2.5 × 10^7^ /kg | 1.14 (0.47-2.79) | 0.760 | 0.78 (0.44-1.37) | 0.396 |
| HLA disparities ≥ 3 | 1.66 (0.66-4.13) | 0.275 | 1.13 (0.64-1.97) | 0.665 |
| Female donor to male recipient | 2.57 (1.05-6.26) | **0.037** | 1.99 (1.12-3.53) | **0.018** |
| TBI 2-4 Gy-based regimens | 2.67 (0.94-7.56) | 0.064 | 1.35 (0.66-2.73) | 0.401 |
| Landmark at 28 days |  |  |  |  |
| High FENa (≥ 1%) at 28 days | 0.86 (0.30-2.48) | 0.792 | 1.02 (0.52-2.00) | 0.936 |
| Age ≥ 45 years | 6.51 (1.38-30.70) | **0.017** | 1.82 (0.92-3.57) | 0.080 |
| HCT-CI ≥ 3 | 1.29 (0.41-4.00) | 0.659 | 0.89 (0.41-1.92) | 0.781 |
| High-risk disease status at CBT | 1.05 (0.39-2.80) | 0.920 | 2.12 (1.14-3.96) | **0.017** |
| Cord blood TNC ≥ 2.5 × 10^7^ /kg | 0.92 (0.36-2.34) | 0.868 | 0.69 (0.39-1.22) | 0.209 |
| HLA disparities ≥ 3 | 1.71 (0.65-4.49) | 0.276 | 1.12 (0.63-1.98) | 0.684 |
| Female donor to male recipient | 2.47 (0.96-6.31) | 0.058 | 1.90 (1.05-3.41) | **0.031** |
| TBI 2-4 Gy-based regimens | 2.56 (0.84-7.71) | 0.094 | 1.24 (0.60-2.57) | 0.552 |

FENa, fractional excretion of sodium; HCT-CI, hematopoietic cell transplantation comorbidity index; CBT, cord blood transplantation; TNC, total nucleated cell; HLA, human leukocyte antigen; TBI, total body irradiation.

The *P*-values in bold are statistically significant (<0.05).
